# Characterization and Inkjet Printing of an RNA Aptamer for Paper-Based Biosensing of Ciprofloxacin

**DOI:** 10.3390/bios9010007

**Published:** 2019-01-02

**Authors:** Jeannine Jaeger, Florian Groher, Jacqueline Stamm, Dieter Spiehl, Johannes Braun, Edgar Dörsam, Beatrix Suess

**Affiliations:** 1 Department of Biology, Technische Universität Darmstadt, 64287 Darmstadt, Germany; jaeger.jea@bio.tu-darmstadt.de (J.J.); groher@bio.tu-darmstadt.de (F.G.); braunj@bio.tu-darmstadt.de (J.B.); 2 Institute for Printing Science and Technology, Technische Universität Darmstadt, 64289 Darmstadt, Germany; stamm@idd.tu-darmstadt.de (J.S.); spiehl@idd.tu-darmstadt.de (D.S.)

**Keywords:** biosensor, aptamer, ciprofloxacin, detection, printing, fluorescence

## Abstract

The excessive use of antibiotics in food-producing animals causes a steady rise of multiple antibiotic resistance in foodborne bacteria. Next to sulfonamides, the most common antibiotics groups are fluoroquinolones, aminoglycosides, and ß-lactams. Therefore, there is a need for a quick, efficient, and low-cost detection procedure for antibiotics. In this study, we propose an inkjet-printed aptamer-based biosensor developed for the detection of the fluoroquinolone ciprofloxacin. Due to their extraordinary high affinity and specificity, aptamers are already widely used in various applications. Here we present a ciprofloxacin-binding RNA aptamer developed by systematic evolution of ligands by exponential enrichment (SELEX). We characterized the secondary structure of the aptamer and determined the KD to 36 nM that allow detection of antibiotic contamination in a relevant range. We demonstrate that RNA aptamers can be inkjet-printed, dried, and resolved while keeping their functionality consistently intact. With this proof of concept, we are paving the way for a potential range of additional aptamer-based, printable biosensors.

## 1. Introduction

The increasing antibiotic resistance of bacteria represents one of the main public health concerns. Excessive use of antibiotics both in therapy and food production is often assumed to be the main cause [1]. An inevitable consequence is that bacteria become more resistant to antibiotics, which renders them ineffective over time. To prevent further spreading, fast, efficient, and cost-effective test systems are therefore important to control food for potential contamination with antibiotics. Biosensors as test systems have already proven themselves in various areas such as food quality control [2].

With the discovery of SELEX (systematic evolution of ligands by exponential enrichment), a technique that allows aptamer development for specific recognition of any target molecule, the possibility for a new type of biosensor has arisen [3,4]. Aptamers are short deoxyribonucleic acids (DNA) or ribonucleic acids (RNA) with a length of 25 to 100 nucleotides. Like antibodies, they can bind their target molecules with high affinity and specificity by forming complex three-dimensional binding pockets that often almost completely enclose their specific target [5].

Biosensors that make use of an aptamer for target recognition are called aptasensors. With respect to their detection system, optical aptasensors can be subdivided into different subclasses: fluorescence (labeled and label-free), colorimetric (e.g., gold nanoparticle aptasensors), chemiluminescence and other optical aptasensors (e.g., surface plasmon resonance aptasensors) [6]. In recent years, several aptasensors that recognize not only antibiotics [7,8,9,10] but also other substances like melamine [11] or proteins [12] have been described. With a share of 19%, fluoroquinolones form a large part of antibiotics used in animal fattening [13]. Therefore, ciprofloxacin (CFX), as a major member of the fluoroquinolone group, is particularly interesting as a new ligand for the development of an aptasensor [13].

Printing of biosensors is a way to easily and rapidly produce a device potentially capable of transferring a biosensing reaction from the test tube in the lab to a sensor for use in the field [10]. If even more components and materials are added in addition to the target-binding molecule itself, more easily readable sensors could be created [14]. Thus, it is possible to use a sensor without any additional lab equipment.

We recently reported the selection of CFX-binding RNA aptamers aimed at the development of new synthetic riboswitches [15]. In addition to the identification of a new CFX-binding riboswitch, several other CFX-binding aptamers could be identified with excellent CFX-binding properties. These aptamers represent outstanding biosensors because fluoroquinolones possess autofluorescence that is quenched after ligand binding [15]. The change in fluorescence intensity directly correlates with the amount of bound CFX. With this simple detection of the ligand, we have a direct detector system that fits our requirements.

Here we exploit the CFX aptamer R10K6_V11 as CFX aptasensor. We performed a detailed structure–function analysis and characterized the fluorescence-based detector system. We then inkjet-printed the aptasensor onto paper-based support material. We analyzed functionality after printing and repeated drying–resolving cycles. The experiments show that the aptasensor can be inkjet-printed without damage and that drying and resolving of aptamers is possible while maintaining their functionality.

## 2. Materials and Methods

### 2.1. In-Line Probing Experiments

The RNA aptamer was dephosphorylated and subsequently 5’ labeled with 32[P]-γ-ATP as previously described [16]. After purification by denaturing polyacrylamide gel electrophoresis, 35 kcpm of 5’ 32[P]-labeled RNA were incubated for 68 h at 22 °C in 10 mM Tris HCl (pH 8.3), 10 mM MgCl_2_ and 100 mM KCl. For a size marker, radioactive-labeled RNA was subjected to alkaline hydroxylation by incubating it for 3 min at 96 °C in 50 mM Na_2_CO_3_ (pH 9.0). In addition, RNA was incubated with 20 U RNase T1 for 3 min at 55 °C to identify guanines [17]. All reactions were ethanol precipitated. Pellets were dissolved in 5 M urea and separated by denaturing polyacrylamide gel electrophoresis. The gel was subsequently dried and analyzed using phosphoimaging (GE Healthcare, Chicago, IL, USA).

### 2.2. Fluorescence Titration Experiments

By measuring the fluorescence quenching as a function of RNA concentration in the presence of CFX or CFX-derivatives, the dissociation constants (KD) for RNA-ligand complexes were determined. The fluorescence intensities were measured on a Fluorolog FL3-22 (Horiba Jobin Yvon). Excitation and emission wavelengths for CFX and each CFX-derivative are given in Table 1.

In preparation for the titration, the RNA solution was heated to 95 °C for 5 min and directly snap-cooled on ice for 5 min to ensure proper folding of the RNA. Afterwards, binding buffer was added to a final concentration of 40 mM HEPES, 125 mM KCl, 5 mM MgCl_2_ (pH 7.4).

All measurements were performed with an integration time of 0.5 s at 25 °C. 50 nM CFX or fluoroquinolone-derivative was mixed with increasing amounts of folded RNA and the fluorescence intensity was measured. Between each step of RNA addition, the solution was stirred for 1 min and equilibrated for 1 min. Curve fitting was done using Prism (GraphPad Software) and nonlinear regression analysis with following equation by least squares fitting: *Y* = B_max_*X^h^/(KD^h^ + X^h^), with *B_max_* = maximum binding, *h* = hill slope, *X* = concentration of RNA. All measurements were performed at least twice.

### 2.3. Inkjet Printing Experiments

The printing experiment consisted of five steps: Aptamer printing, drying, CFX application, redrying, and rehydration. The printing system Autodrop (Microdrop Technologies, Norderstedt, Germany) was used for printing varying amounts (0.25, 0.5, 1, 2, 5 µL) of 26.67 µM RNA into 1 cm^2^ squares onto the paper-carriers. The filter paper Whatman Grade 1 (GE Healthcare) was chosen for its weak autofluorescence at the excitation and emission peaks of CFX [18]. The deposited fluid volume was controlled by setting the space between consecutive drops (drop space) and the number of drops along one edge (grid size). Together with the analyzed single drop volume of 310 pL, the resulting volume can be calculated in µL or mL/m^2^. When combined with 5 µL 10 µM CFX solution, a ratio of aptamer to CFX can be calculated. The parameters used for the experiments are given in Table 2. Each sample was printed three times.

After printing, the samples were dried at room temperature for at least one day. One set was dried and stored for eight days before applying CFX. The quenched fluorescence of the applied CFX onto the printed aptamer squares was measured using the imager Fusion FX Edge by Vilber Lourmat (Collégien, France). The best fitting excitation illumination (peak at 312 nm) and absorption filter (470–590 nm) were close to the excitation and emission peaks of the analyzed fluoroquinolones. As the UV-transilluminator is fitted with parallel tubes that produce inhomogeneous illumination, each sample set was introduced separately at the same spot along the center tube.

Afterwards, the samples were stored again and re-dried at room temperature. After one week, the spots were rehydrated with 5 µL drops of Millipore Milli-Q (MQ) water and pictures were taken with the imager before and afterwards.

## 3. Results

### 3.1. Probing the Secondary Structure of the CFX Aptamer R10K6

The 103 nt long CFX-binding RNA aptamer R10K6 was previously selected [15]. R10K6 showed exceptional ligand binding with a reported KD of 31.2 nM. Consequently, the aptamer was perfectly suited to be developed into a highly efficient CFX aptasensor.

**Figure 1:**
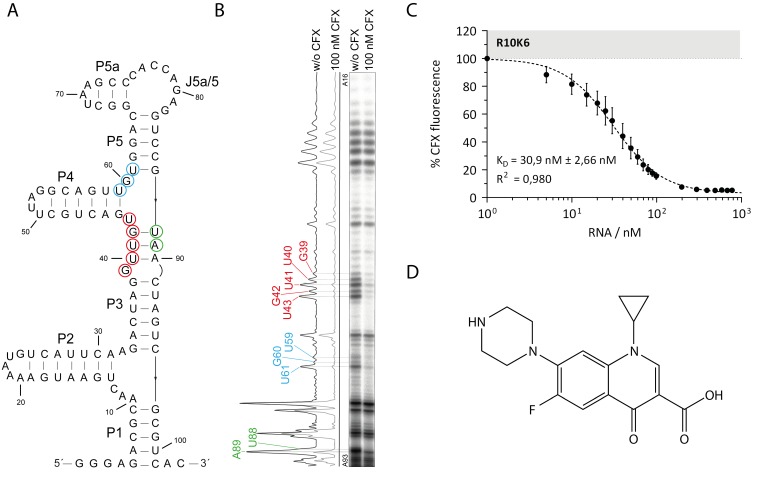
Biochemical characterization of the ciprofloxacin (CFX) binding aptamer R10K6. (**A**) Predicted secondary structure of R10K6 by mfold [19,20,21], the six stem regions and one single-stranded region are marked with P1-P5a and J5a/5, respectively. Nucleotides with changes in the cleavage pattern are encircled and color-coded. (**B**) In-line probing experiment of the CFX binding aptamer R10K6. The cleavage pattern and the respective quantification is shown in the absence (w/o CFX) and presence (100 nM CFX) of CFX. Regions with changes in the cleavage pattern are marked and color-coded. (**C**) Fluorescence titration spectroscopy for R10K6 with CFX as ligand. The experiment was performed three times and mean and standard deviation are shown respectively (**D**) Chemical structure of CFX [22].

The secondary structure of the aptamer was predicted by mfold ([19,20,21], Figure 1) and confirmed by in-line probing. In-line probing makes use of the self-cleaving ability of ribonucleic acids. Flexible regions will be cleaved with higher probability than less flexible ones. Consequently, double stranded parts or regions involved in tertiary interaction or ligand binding are being protected. By using in-line probing, we were able to confirm the predicted secondary structure of R10K6 (Figure 1B). The aptamer folds into six stems P1-P5a connected via two three-helix junctions and a single stranded region between P5 and P5a, J5a/5. Three regions with significant CFX-dependent changes in the cleavage pattern indicate their role in ligand binding (G39-U43, U59-U61 and U88-A89, marked in red, blue and green, respectively, in Figure 1A,B). It defines the CFX binding pocket composed of 10 nts.

### 3.2. Ligand Binding Specificity of R10K6

The chemical structure of CFX is shown in Figure 1D. As all fluoroquinolones, it consists of a quinoline carboxylic acid backbone. Upon binding to the aptamer, the intrinsic fluorescence of CFX is quenched. By measuring the decrease of the fluorescence as a function of RNA concentration in the presence of CFX, a dissociation constant (KD) for the RNA-ligand complex of 31 nM was determined.

**Figure 2:**
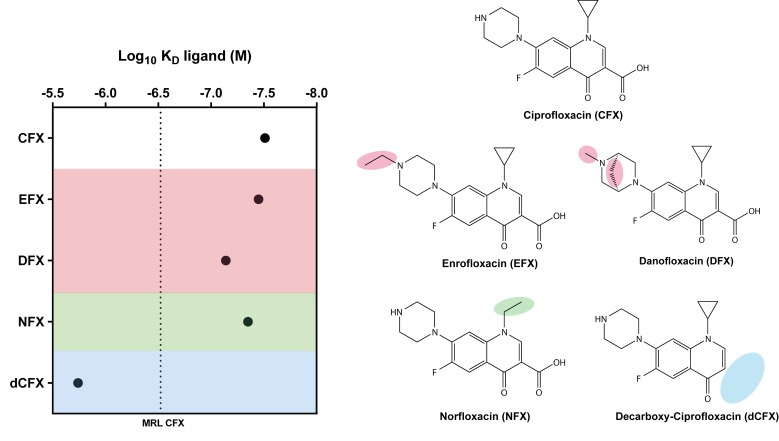
Specificity of the molecular recognition of the CFX-binding aptamer R10K6 and its impact on the dissociation constant. Left: Plot of the Log10KD-values (M) of CFX and CFX-derivatives. The maximum residue limit (MRL) for CFX in milk samples is marked as a dotted line [23,24]. Right: Chemical structure of CFX and CFX derivatives. Changes relative to CFX in the chemical structure are shaded.

Fluorescence titration spectroscopy was then performed with different fluoroquinolone derivatives to analyze the influence of the different side groups on the binding behavior. In Figure 2, the logarithmic KD values for each ligand are displayed. Enrofloxacin (EFX), having a modification on the piperazinyl residue, and norfloxacin, (NFX) having changes on the cyclopropyl residue located on N1, showed a similar KD as CFX. Danofloxacin (DFX), also with modifications on the piperazinyl residue, showed only slightly reduced binding compared to CFX. The KD-value of decarboxy ciprofloxacin (dCFX) with a deletion of the carboxyl group on C3 clarifies the importance of this side group for CFX binding. This indicates that the aptamer has a rather broad binding specificity for fluoroquinolones.

The maximum residue limit (MRL) for CFX in food samples, e.g., milk, is approximately 300 nM (100 ppb) [23,24] (marked as a dotted line in Figure 2). Thus, the limit is about 10-fold higher than the determined dissociation constants of CFX, EFX, DFX, and NFX. Consequently, not only CFX but also the other tested compounds could be detected with the aptasensor. As demonstrated, R10K6 is an aptamer that is perfectly suited as biosensor for detection of fluoroquinolones in food samples due to its high binding specificity and affinity for CFX and CFX-derivatives. 

### 3.3. Defining a Minimal CFX-Binding Aptamer

**Figure 3:**
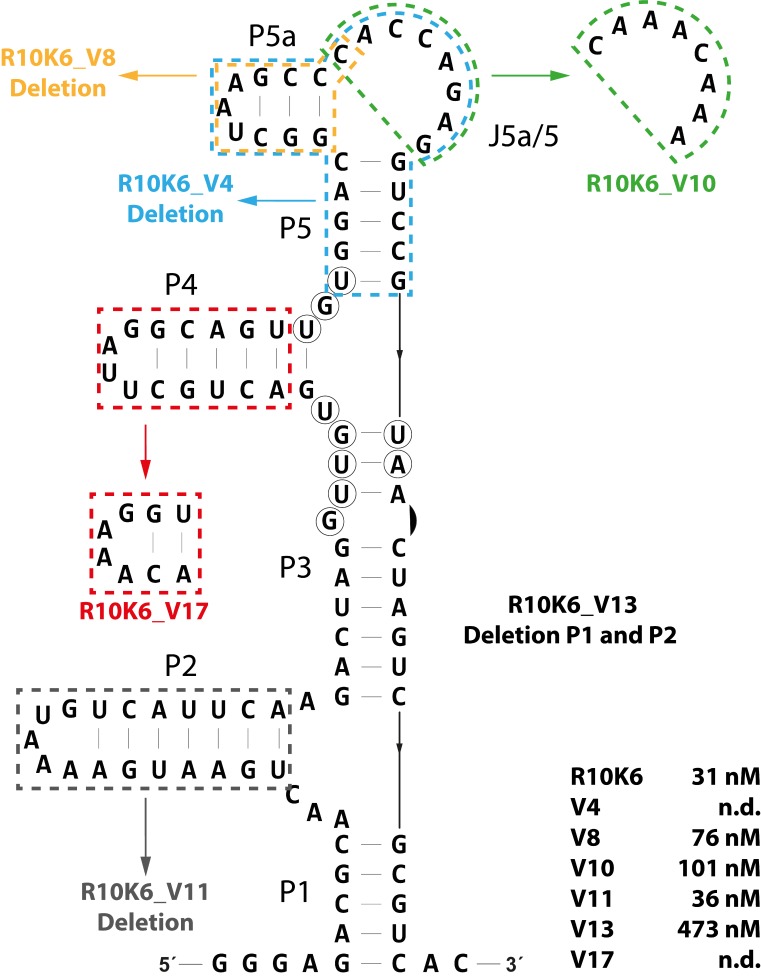
Mutational analysis the CFX-binding aptamer R10K6. Mutations introduced to study the structure–function relationships are indicated. The respective dissociation constants of the mutations are given below.

For robust application as a biosensor, it is advantageous if the aptamer is present in a minimal and compact structure and areas that are not necessary for binding are removed. We therefore designed mutants in which different regions of the aptamer had been deleted and determined their remaining binding capacity. The results are summarized in Figure 3.

The complete removal of stem P5 and P5a (mutation V4) resulted in a complete loss of CFX binding. In all likelihood, parts of the CFX binding pocket are destroyed with this mutation, especially nucleotide U61. Previously, U61 was identified as part of the major binding pocket by in-line probing (Figure 1A,B). However, the deletion of only stem P5a or changing the sequence of the single stranded region J5a/5 between P5a and P5 also significantly reduced ligand binding (V8: 76 nM, V10: 101 nM). This indicates that although there is little evidence of a role in direct ligand binding, these regions still have stabilizing effects on the aptamer. Therefore, we decided not to change the upper part of the aptamer. The mutation V17, in which P4 was shortened but closed with a stabilizing tetraloop, no longer showed any binding, suggesting that parts of P4 are involved in stabilizing the aptamer rather than in ligand binding. In addition, the in-line probing results (Figure 1A,B) confirmed that P4 does not belong to the binding pocket, consisting of 10 nts. Deletion of the complete lower region (∆P1∆P2, V13) resulted in a dramatic loss of binding activity (KD = 473 nM), whereas the deletion of only P2 did not affect the dissociation constant at all (KD = 36 nM). It indicates that P1 seems to play an important role in stabilizing or structuring the aptamer.

Finally, the R10K6_V11 was revealed as the most functional mutant. It represents a shorter version of the aptamer R10K6 without loss of ligand binding affinity. In comparison to R10K6, R10K6_V11 is shortened by 19 nts and has a final length of 84 nts. The determined dissociation constant of 36 nM for V11 is similar to the original full-length aptamer R10K6 (31 nM).

### 3.4. The CFX Aptamer Can Be Printed and Retains Functionality

In the next step, we performed inkjet-printing experiments to build a paper-based detector platform for CFX. The sensor principle relies on quenching of the autofluorescence of CFX after being bound by the aptamer. A sensor usually produces a signal when the target is detected. In this case, the detection of the target was associated with a decrease of the signal. Hence, a washing step would be counterproductive as our detection was based on measuring the fluorescence of the unbound CFX.

The aptamer solution was printed onto a defined location and the CFX solution was pipetted on top. As unbound CFX will fluoresce, the ratio between aptamer and CFX molecules was varied by printing increasing amounts of the aptamer solution and keeping the CFX concentration and amount constant. This way allowed us to find the ideal ratio, where most of the CFX molecules are bound by the aptamer. For evaluation, we considered four different scenarios. The first scenario represented the ideal situation: the fluorescence intensity was measured directly after CFX application in a wet state. But we were also interested in investigating storage options after application, for example when there is a need to mail the used sensor to a lab for further analysis. There, it might be investigated in its dried state or after rehydration. After storage of seven days, the used sensor is in a dry state. We expected that the evaluation would yield incorrect results on a dried sensor and wanted to try to rehydrate the spots by applying 5 µL of water to each spot in the third scenario. A fourth test took the storability of the printed aptamer into account by storing a printed sample for eight days and then testing its CFX-binding in a wet state.

**Figure 4:**
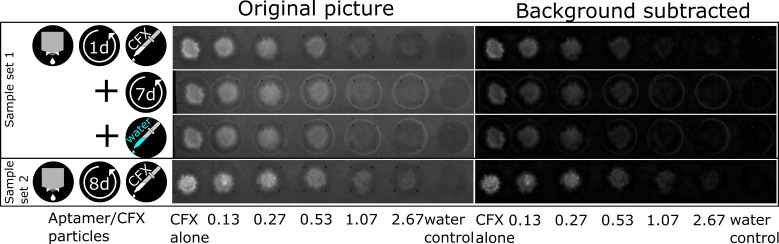
Fluorescence pictures of the printed CFX biosensors. The first three rows show the first sample set in the states wet (dried for one day after printing of the aptamer, then CFX was added), dry (drying for seven days after the measurement in the first row) and rehydrated (by adding water after the measurement in the second row). The last row shows the second sample set in the wet state (dried for eight days after printing of the aptamer, then CFX was added). Shown is the original picture as taken by the imager on the left and the background subtracted one on the right. The abscissa shows the ratio of aptamer to CFX. Each set was printed and evaluated three times, but only one is shown.

In more detail, a solution of 26.67 µM aptamer R10K6_V11 was printed into 1 cm^2^ squares onto filter paper. By using different distances between printed drops and hence different numbers of drops for each square, 0.25, 0.5, 1, 2, and 5 µL of the aptamer solution were added (see Table 2). The samples were stored at room temperature for one (sample set 1) or eight days (sample set 2). Each sample set consisted of five squares of different aptamer amounts and two empty spaces for the references of pure CFX and pure water. During the antibiotic application, 5 µL of a 10 µM CFX solution were added by pipetting onto each square and the CFX reference spot. For the water reference, 5 µL of water was pipetted. The fluorescence was measured in different states. Sample 1 was measured in wet, dry (after 7 days of drying), and rehydrated state, sample 2 only in the wet state. The pictures taken with the imager for evaluation are shown in Figure 4 (camera settings: aperture = 4; exposure time = 520 ms). Each set was printed and evaluated three times.

**Figure 5:**
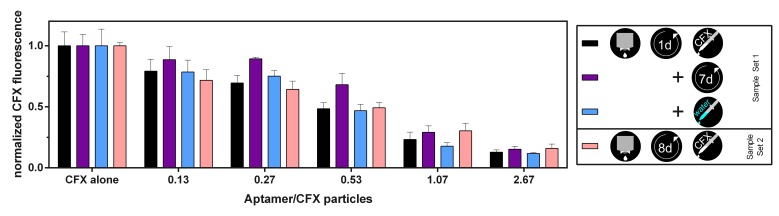
Evaluation of the printed CFX biosensors shown in Figure 4. The quenched fluorescence is shown after subtraction of the water reference and normalization to pure CFX, for sample 1 in wet, dry and rehydrated state and sample 2 only in wet state. Error bars represent standard deviation over three samples.

To analyze the intensity change, the background was subtracted from the pictures with an image editing software. The raw integrated density was evaluated with 100 pixel diameter circles placed over the samples. The outside circles, which appear after drying, are not included, because they also occur in the water reference. The reason for these circles could be a dissolution of additives in the paper contributing to a coffee ring effect during their drying. The raw data was processed by subtracting the water reference and normalizing to the CFX reference. Figure 5 shows the quenching ability of the printed biosensors for increasing aptamer to CFX ratio and for the different states. The smaller the normalized CFX fluorescence, the more CFX was bound and quenched. All experiments showed a decrease in fluorescence for increasing aptamer to CFX ratios, but with some variations. Comparing the different samples, sample 1 performs best, but also the other states show excellent quenching abilities and there is no significant differences observable. A further increase in the amount of aptamer only lead to a slight increase in quenching. After drying this sample for seven days, the fluorescence increased, which could be due to a decreased functionality of the aptamer in the dried state. After rehydration of this sample, original fluorescence, equivalent to CFX-binding capability, is almost completely restored. Sample set 2, which tested a sensor dried for eight days after printing the aptamer, showed the same fluorescence after adding CFX (within the scope of the error) as sample set 1 in its first measurements. This indicates that storage in the dried state for this duration has no significant influence on the binding capability of the aptamer.

By using several aptamer to CFX ratios, we were able to analyze the binding ability and functionality of the aptamer after drying and rehydration. With a 1:1 ratio of aptamer to CFX molecules, the quenched fluorescence in the wet state was already reduced to 23 ± 7% of the pure CFX fluorescence, meaning that most aptamers were able to bind. With higher aptamer concentrations, the fluorescence ratio decreased only slowly. The results demonstrate that an RNA aptamer can be printed on paper-based support material and is still able to bind its ligand after drying.

## 4. Conclusions

In this study, we characterized a short version of a CFX-binding aptamer that was able to recognize a wide range of fluoroquinolones with high affinity. We defined a 10 nt long region that forms the ligand binding pocket. The analysis of CFX derivatives allowed us to investigate which specific side groups are bound by the aptamer, and which not. The titration experiments revealed that single changes in side-groups (on piperazinyl residue or cyclopropyl residue) did not have a major impact on ligand binding. Moreover, the experiments revealed that especially the carboxyl-group (dCFX) on C3 is important for CFX binding.

Since the European MRL for CFX in milk is about 300 nM (100 ppb) [23,24], the aptamer R10K6_V11 should be able to detect CFX contaminations. Consequently, the aptamer is suitable as a biosensor for food samples like milk, due to its high binding affinity for CFX and a multitude of its derivatives. However, we should test the stability of our RNA aptamer due to the presence of RNase in biological samples like milk.

In addition, we were able to build a paper-based detector platform. We found that the aptamers, which were printed onto filter paper and dried overnight, still retained their functionality of quenching the fluorescence of CFX. After repeated drying of the sample, fluorescence faded slightly and increased again after rehydration of the spots with water. Evidently, fluorescence is strongest while wet and should be measured in a wet state. Another sample, which was stored for a week after printing, showed the strongest fluorescence after CFX application. This indicates that some functionality of the aptamers is lost during storage on paper at room temperature. Different storing conditions should be investigated in the future and could improve the functional integrity.

In sum, inkjet printing of RNA-based aptamers allowed us to build a simple detector platform for the detection of CFX on paper-based support material that makes use of a high affinity CFX-binding aptamer. The results demonstrate that an RNA-based aptamer can be printed and dried without loss of functionality. This proof of concept forms the basis for a wide range of potential RNA-aptamer based biosensors on paper-based support material.

## Figures and Tables

**Table 1:** Excitation and emission wavelengths of fluoroquinolones and derivatives used in this study.

Fluoroquinolone	Excitation (nm) *	Emission (nm) *
Ciprofloxacin (CFX)	335	420
Danofloxacin (DFX)	325	425
Decarboxy-Ciprofloxacin (dCFX)	320	420
Enrofloxacin (EFX)	330	420
Norfloxacin (NFX)	330	425

* 5 nm slit width.

**Table 2:** Printer Settings.

Dropspace/mm	Grid Size	mL/m^2^	µL	Aptamer/CFX Particles
0.253	28	5	0.25	0.13
0.177	40	10	0.5	0.27
0.124	57	20	1	0.53
0.088	80	40	2	1.07
0.056	127	100	5	2.67
